# Production of natural colorants by liquid fermentation with *Chlorociboria aeruginascens* and *Laetiporus sulphureus* and prospective applications

**DOI:** 10.1002/elsc.202000079

**Published:** 2021-01-26

**Authors:** Marlen Zschätzsch, Susanne Steudler, Olena Reinhardt, Pia Bergmann, Franziska Ersoy, Stephanie Stange, André Wagenführ, Thomas Walther, Ralf Günter Berger, Anett Werner

**Affiliations:** ^1^ Institute of Natural Materials Technology Chair of Bioprocess Engineering Faculty of Mechanical Engineering Technical University of Dresden Dresden Germany; ^2^ Institute of Natural Materials Technology Chair of Wood Technology and Fibre Materials Technology Technical University of Dresden Dresden Germany; ^3^ Institute of Food Chemistry Gottfried Wilhelm Leibniz University Hannover Hannover Germany

**Keywords:** *Chlorociboria aeruginascens*, xylindein, *Laetiporus sulphureus*, laetiporic acid, natural dye

## Abstract

The replacement of potentially hazardous synthetic dyes with natural dyes and pigments are of great interest for a sustainable economy. In order to obtain cost‐efficient, environmentally friendly and competitive products, improvements in the cultivation and extraction of pigment‐producing organisms and in dyeing processes are necessary. In our study, we were able to scale up the production of xylindein by *Chlorociboria aeruginascens* from 3 to 70 L bioreactor cultivations. We have identified important bioprocess parameters like low shear stress (150 rpm, tip speed <0.5 m/s) for optimal pigment yield (4.8 mg/L/d). Additionally, we have demonstrated the potential of laetiporic acid production by *Laetiporus sulphureus* in various cultivation systems and media, achieving dried biomass concentrations of almost 10 g/L with a 7 L bioreactor cultivation after 17 days. Extractions performed at 70°C and 15 min incubation time showed optimal results. To the best of our knowledge, we have described for the first time the use of this pigment in silk dyeing, which results in a brilliant hue that cannot easily be produced by other natural pigments.

**Abbreviations**
DCM: dichloromethane
MEK: methyl ethyl ketone
OD: optical density

## 1 INTRODUCTION

Colors play a special role in our everyday life. Already in the middle of the 19th century the synthetic dye industry emerged [1]. Further advances in chemical synthesis replaced previously used colors made from natural raw materials. Synthetic dyes have a petrochemical origin and certain azo dyes have been shown to pose harmful health effects. Even more alarming is that their production and use often cause environmental hazards around the textile industry through the release of toxic substances, especially in developing countries. In order to improve environmental conditions and provide the prerequisites for a circular bio‐economy, research is needed to advance the industrial production of natural dyes [2].

Various natural sources have been used for dyeing before the synthetic era. For example, inorganic mineral pigments like ultramarine and malachite exist next to dyes from biological origin. Main sources are plants, algae, microbes, and animals like cochineal. Well know examples are alizarin from madder plant, phycocyanin from cyanobacteria, and red pigments from *Monascus* species (Ascomycota) [3, 4]. Although natural dyes have considerable ecological advantages over synthetic dyes, their industrial production has remained difficult until today, mainly due to low yields [5]. There are also disadvantages in the production of colors from plants, such as seasonal fluctuations or the conflict with the food industry, especially with regard to the growing area.

The group of fungi holds great potential to exploit their natural dyes or pigments, which represent an interesting alternative to other natural sources [6]. These pigments exhibit several key properties, for example, colorfastness [2] and ecological production using agricultural by‐products, which contributes to a circular bioeconomy. Additionally, fungal dyes and pigments as secondary metabolites have an immense chemical variation. Besides the color appearance, they can exhibit antibacterial, anticarcinogenic, insecticidal, fungicidal (mycotoxins), or antioxidant properties [7, 8]. However, the fungal potential as high value metabolite producer has hardly been used so far.

**PRACTICAL APPLICATION**

The production of clothing, art, and many everyday objects involves the use of dyes and pigments. Replacing synthetic colors with natural colorants contributes to a sustainable economy. Important steps in this direction require the identification of optimal cultivation parameters of pigment producing organisms and improvements in pigment extraction and the subsequent application process. Using bioprocess engineering, we identified important parameters for pigment production by the two fungi *Chlorociboria aeruginascens* and *Laetiporus sulphureus*, producing the blue‐green xylindein and the orange‐yellow laetiporic acid pigments, respectively. We could demonstrate the coloration of wood by xylindein and textile dyeing by laetiporic acid. The transfer of the identified culture parameters to industrial scale for xylindein and further scale up and dyeing experiments for laetiporic acid will broaden the application of natural colors.

Fungal species capable of producing pigments are divers and comprise Ascomycota and Basidiomycota. Two examples are xylindein producing *Chlorociboria aeruginascens* and the pigment family of laetiporic acids produced by *Laetiporus sulphureus*. Xylindein can be found in historical intarsia work. Since the 15th century wood colored by *Chlorociboria* sp. has been used for these artworks [9, 10]. Recently, especially the group around Sara C. Robinson [11, 12] investigated the production of xylindein by cultivating *Chlorociboria* sp. to produce biomass and thereby achieved high xylindein yields [13]. Next to solid state fermentation, addition of wood substrates to agar plates or liquid cultures using malt extract medium have been used for cultivation [14, 15, 16]. Our previous study identified orange juice in liquid cultures as a suitable substrate for xylindein production [17]. Furthermore, we could show that xylindein formation is triggered by nitrogen limitation [17]. Organic solvents such as dichloromethane (DCM) or chloroform can be used for pigment extraction [18, 19]. The optimization of xylindein extraction might allow to further advance the natural dyeing of textiles, which has already been shown for several materials, for example, cotton, wool, and polyamide [12, 20]. Application of xylindein as paint color was accomplished using DCM extracts and native oils, especially raw linen oil, have been identified as suitable carriers for xylindein [21]. However, a successful transfer to paints was not yet possible [22]. Xylindein is not only promising for mycological wood discoloration and textile dyeing, but also as a fluorescent marker or an organic semiconductor [13, 23, 24]. Of particular interest are also the approaches of Maeda et al. [25] to use xylindein for medical and pharmacological applications. So far, xylindein can only be purchased in small quantities through the working group around Sara C. Robinson [26]. The production of xylindein by large‐scale liquid cultivation is a prerequisite for the industrial use of the pigment.

Laetiporic acid has been described in literature as a polyene for the first time in 2004 [27], with the identification of several variants 1 year later [28]. These laetiporic acids therefore form a whole family of non‐carotenoid orange‐yellow polyene pigments. Stirred bioreactor cultivations have been described, but the mentioned studies concentrated on pigment identification and the insulinogenic properties of the produced extracellular polysaccharides. Coloration of the fungal mycelium was not mentioned, but an optimal mycelium growth at a very acidic pH of around two was identified [29]. A recent study showed antifungal effects by *Laetiporus* polyenes [8]. To the best of our knowledge, these pigments have not been produced for subsequent use as natural colorants.

In the present study, we investigated the cultivation of *C. aeruginascens* and *L. sulphureus* in bioreactors up to 70 L with respect to enhanced pigment production. Furthermore, we analyzed parameters for pigment extractions and the possible application of the pigments in wood coloring or fabric dyeing.

## 2 MATERIALS AND METHODS

### 2.1 Microorganism and cultivation

*C. aeruginascens* (A 39 provided by IHI Zittau, Germany) was maintained on 50% (v/v) orange juice agar plates (50% orange juice from Sonniger®, 100% fruit content, manufactured for ALDI Nord, Germany; 30 g/L Agar‐Agar, Roth, Germany). For submers cultivation, two 1 cm^2^ of a 14‐day‐old fungal agar culture were transferred to 150 mL 5% (v/v) orange juice medium (orange juice from Sonniger®, ALDI Nord, Germany; 22°C, 120 rpm, 14 d) according to Stange et al. [19]. For the fermentation experiments, 200 mL of submers culture (120 rpm, 14 days) was used to inoculate 2 L 5% (v/v) orange juice medium containing 1 mL/L silicone antifoam emulsion (Roth, Germany) in a 3 L bioreactor (Z611000310, Applikon, Netherland). These were cultivated at 22°C and 150 rpm for 14 days. Every 2 days a sample (5 mL) was taken. For the scale up experiments, 500 mL or 5 L of submers culture was used to inoculate 5 or 55 L 5% (v/v) orange juice medium containing 1 mL/L antifoam in a 7 L (Z611000720, Applikon, Netherland) or 70 L bioreactor (Z620003070, Applikon, Netherland), respectively. The cultivations were carried out at 22°C with 150 rpm and an aeration rate of 0.5 vvm (7 L bioreactor) or 100 rpm and 0.27 vvm (70 L bioreactor). Samples were taken every 2 days (5 mL, 7 L bioreactor) or every day (50 mL, 70 L bioreactor). The equipment of the reactors is summarized in Table S1.

*L. sulphureus* (DSMZ 11211, Germany) was maintained on 1.5% (w/v) agar plates with standard nutrient liquid (SNL) medium at 24°C. Information on media composition see Section 2.2 and Supporting Information. For submerse cultivation, 1 cm^2^ of a 7‐day‐old agar plate was transferred to 200 mL medium. Cultures were harvested after 14 days. For the fermentation experiments, 5% (v/v) submers culture was used to inoculate 4 L Moser b medium in a 7 L bioreactor (Z611000720, Applikon, Netherland). These were cultivated at 26°C with an agitation rate of n = 300 rpm and an aeration rate of pO_2_ = 1 or 2 L/min. A sample (10 mL) was taken every weekday.

### 2.2 Media optimization for *L. sulphureus* cultivation

Standard media like SNL and Moser b, both containing 30 g/L glucose and trace elements (content see supporting information) have been tested for mycelium cultivation in shaking flasks (170 rpm, 26°C) and compared to simple media like PDB (potato dextrose broth, Roth, Germany) containing 13.25 g/L broth and PDB supplemented with 5 g/L ground beech (<0.5 mm).

### 2.3 Characterization of 3 L bioreactor system cultivating *C. aeruginascens*

For the characterization in the 3 L bioreactor (Z611000310, Applikon, Netherland), the working volume (0.5 to 2 L), aeration rate (0.5 to 2 vvm). and stirrer speed (0 to 800 rpm) were varied. The experiments were performed for water, culture medium, and culture medium with antifoam (1 mL/L, Silicone antifoam emulsion, Roth, Germany).

The *k*_L_a value was determined using the dynamic method by alternating air and nitrogen in double determination. The calculation of the *k*_L_a (oxygen transfer coefficient [h^–1^]) was performed by integrating Equation (1) with CO_2,L_, (oxygen concentration [%]) and $CO_{2,L}^{*}$ (saturation concentration [%]) in the liquid.

(1) $$\frac{dCO_{2,L}}{dx}=k_{L}\;a\cdot \left(C{O_{2,L}}^{*}-CO_{2,\;L}\right)$$

The mixing time was determined using the decolorization method in triple determination. Water was stained blue using 2.5 mL/L starch and 0.5 mL/L iodine potassium iodide solution. Then, 1.25 mL/L sodium thiosulfate was added and the time measured until the solution was “completely” decolorized.

The energy input was calculated according to Equation (2), neglecting the Newton number, with *P*_V_ (volume‐related energy input [W/m^3^]), *V*_reactor_ (working volume of the reactor [m^3^]), *P*_ez‐control_ (energy input [W]), *ρ_Fluid_* (density of the medium [kg/m^3^]), N (stirrer speed [rpm]), D (stirrer diameter [m]), *Np* (stirrer factor 6 for Rushton turbine [‐]) and n (number of stirring blades [‐]).

(2) $$P_{V}=\frac{P_{ez-control}}{V_{reactor}}\;=\frac{\rho_{fluid}\cdot {\left(\frac{N}{60}\right)}^{3}\cdot D^{5}\cdot N_{p}\cdot n}{V_{reactor}}$$

The calculation of the tip speed was based on stirrer diameter and speed according to Equation (3) with *v*_Tip_ (tip speed [m/s]), *N* (stirrer speed [1/min]) and D (diameter of the stirring blade [m]).

(3) $$v_{Tip}=\frac{N}{60}\cdot \pi \cdot D$$

### 2.4 Process analytics

The *C. aeruginascens* biomass concentration was determined gravimetrically. For this purpose, biomass suspensions (2 mL using 3 or 7 L bioreactor, 40 mL using 70 L bioreactor) were separated via a 0.45 μm filter and dried at 103°C. To determine the biomass dry weight of *L. sulphureus*, 5 mL sample was centrifuged (10 min, 5000 g, 25°C), the pellet was washed once and then dried O/N. The reducing sugars were determined by the method of Miller [30]. In addition, the sugar composition was determined by HPLC (Rezex™ RPM Monosacharide Pb^+2^ column, Phenomenex, USA) at 85°C and a flow rate of 0.6 mL/min. The total nitrogen (TN) was determined by disintegration with the Laton total nitrogen cuvette test (LCK338, Hach‐Lange, Germany). The determination of the total organic carbon (TOC) was determined by the difference method of the TOC cuvette test (LCK338, Hach‐Lange, Germany). Freeze‐drying (Christ, Germany) was performed after centrifuged biomass was frozen at –80°C O/N.

### 2.5 Extraction process for xylindein

Disruption of the fresh biomass was carried out using a high‐pressure homogenizer at 2.6 kbar in the French Press (Constant Cell Disruption System TS 6, Constant Systems LTD, UK). Alternatively, the harvested biomass was gently dried at 60°C and then ground in an ultracentrifugal mill (ZM 200, Retsch GmbH, Germany). For solvent screening, 1 mL of pretreated fresh biomass was incubated with 3 mL solvent (30 min, overhead shaker; 1 h, still state). After centrifugation for 5 min at 2600 g (Heraeus Biofuge stratos, Thermo Fisher Scientific GmbH, Germany), the absorption spectrum (300–800 nm) of the supernatant was recorded with a spectrophotometer (Beckman DU 640, Beckman, USA). The following solvents were examined: acetone, acetylacetone, benzyl alcohol, 2‐butanone (methyl ethyl ketone, MEK), dichloromethane (DCM), 1,2‐dichloroethane, dimethyl sulfoxide (DMSO), ethanol, ethyl acetate, glacial acetic acid, isopropanol, methoxypropanol, n‐hexane, phenol, 1,2‐propanediol, toluene, and water. The following parameters for fresh biomass were examined: ratio of solvent (DCM) to extraction material of 1:1, 1:2, 1:3, 1:4; and extraction time of 5, 10, 30, and 60 min (overhead shaker, solvent ratio 1:1), both treated after extraction as described above. In comparison, 10 mg dried biomass was incubated with 3 mL MEK or DCM. The contact time of solvent and extraction material was varied (0.5, 1, 1.5, 2, 2.5, 3, 4, and 5 h). The extraction material was centrifuged (5 min at 2600 g) and the absorption spectrum of the supernatant determined. Analysis of extraction cycles is described in the supporting information.

To determine the temperature stability, both the purified pigment and fresh biomass were dried at 40, 50, 60, 70, 80, and 103°C. A photo documentation of the samples was made after 30, 60, 120, 180 min, and 24 h. To assess the storage stability, both the xylindein powder and two extracts (xylindein solved in DCM or MEK) were examined. All materials were stored in a cold room (8°C) and checked regularly over a period of 9 months and documented.

### 2.6 Extraction process for laetiporic acids

For bioreactor experiments, 1 mL sample was centrifuged (10 min, 5000 g, 25°C), washed with dest. H_2_O and centrifugation was repeated. Biomass was ground and resolved in 10 mL absolute ethanol (99.8%, Roth, Germany). After centrifugation (5000 g, 10 min) the absorption spectrum of the supernatant was recorded with a spectrophotometer (Beckman DU 640, Beckman, USA). For media optimization experiment, the culture was homogenized using an ultra‐turrax (IKA, Germany). Then, 2.5 mL sample was incubated with 2.5 mL absolute ethanol and absorbance of extract was determined as described before.

For extraction optimization several parameters were analyzed. Pretreatment always involved homogenization of the sample. Extractions with 100% solvent were carried out with 7.2 g/L fresh biomass or 0.36 g/L freeze‐dried biomass. Biomass was centrifuged (5000 g, 10 min), the pellet resuspended in solvent (ethanol, methanol, acetonitrile) and incubated for 30 min. After centrifugation (5000 g, 10 min) the absorption of the supernatant was determined. For extractions with 50% (v/v) ethanol biomass was diluted 1:2 with solvent to achieve a final concentration of 7.2 g/L (fresh) or 0.36 g/L (freeze‐dried). Extraction temperatures of 25, 50, 70, and 90°C and extraction times of 15, 30, 45, and 60 min were tested with freeze‐dried biomass (0.36 g/L). For stability experiments, extracted solutions (freeze‐dried, 50% ethanol, 30 min at 70°C) were kept at room temperature and absorbance was determined after 1 h, 2 h, 1 d, and 7 d. Extract stability was also assessed for 50% acetone and 50% acetonitrile after 3 days at room temperature. Color stability of laetiporic acid extracts at different pH values was determined according to Stange et al. [19]. The extract was diluted 1:10 in different pH buffers. Due to this dilution, an extract of 3.6 g/L freeze‐dried biomass (10x higher concentrated) was used. After an incubation of 10 min the absorbance was measured. All extractions have been performed in duplicate and data points represent the mean ± SD.

### 2.7 Application of xylindein (veneer dyeing) and laetiporic acids (silk dyeing)

Three samples each of birch veneer (*Betula sp.)* and beech veneer (*Fagus sylvatica*) with a diameter of 88 mm (veneer thickness 0.9 mm, tangential section) were cut and then soaked in tap water for 10 min and autoclaved at 103°C for 30 min. Additionally, six cellulose filter papers were autoclaved. Under sterile conditions, 50 mL mycelium suspension (liquid culture as described before) was transferred to a filter paper and covered with a veneer (area‐related dry biomass concentration 1.6 mg_BM_/cm^2^). These samples were then transferred to a petri dish (Ø 92 mm) and incubated at 23°C ± 2°C for 12 weeks in the dark. Regular microscopic examination using a binocular (40 × magnification) was performed.

For the application of laetiporic acids, 50 or 200 mg/mL biomass (fresh, centrifuged) diluted in 50% ethanol were treated with an ultra‐turrax. Silk samples (2 cm^2^) were dyed immediately with the complete ethanol‐mycelium mixture individually in conical flasks (50 mL) at 70 or 90°C for 60 min in a thermomixer (Eppendorf, Germany).

## 3 RESULTS AND DISCUSSION

### 3.1 Fermentation of *Chlorociboria aeruginascens* in bioreactors up to 70 L

In our previous studies, we have identified optimal growth parameters using 12‐well plates and shaking flask cultures. The following parameters were retained: liquid fermentation (bubble column or stirred reactor), batch process, 5% orange juice as medium, pH 3.5–5.0 and temperature 20 ± 2°C.

For the scale‐up, various bioprocess engineering parameters have been analyzed using a 3 L bioreactor screening. This included the consumption of nutrients (carbon source, nitrogen source, etc), mixing time, *k*_L_a value as a measure for oxygen input, as well as energy input and stirrer tip speed (measure for shear stress) calculated from stirrer speed, reactor and stirrer geometry.

A fast mass transfer and a good oxygen supply are of elementary importance to avoid unwanted limitations and require rapid mixing and sufficient *k*_L_a values. However, the challenge is to find the right balance between mixing, aeration, and fungal growth as well as product formation. The aim was therefore to identify an optimum between mycelium growth and the necessary bioprocess parameters. For this purpose, the bioreactor was first characterized in different modes. For shear‐sensitive organisms, the use of bubble columns has proven successful. To simulate a bubble column, the 3 L reactor was operated without stirring. The *k*_L_a values were in the range of 7–20 h^–1^ depending on the aeration rate (0.5–2.0 vvm) and the working volume (0.5–2 L). The mixing times were in the range between 5 and 15 s. For the characterization of the 3 L bioreactor with installations and agitator, a moderate aeration rate of 1 NL/min was chosen. In Table 1 the different parameters are depicted. As expected, by increasing the stirrer speed (150–800 rpm), the *k*_L_a values increased and reached a maximum of more than 110 h^–1^. The mixing time played a rather minor role in the stirred version and was in the range of 1–6 s, which indicates short mixing times and good mixing. Subsequently, the tip speed and the power input were determined on the basis of the selected parameters and the reactor geometry.

**TABLE 1:** Overview of the detected scale‐up criteria as a function of the stirrer speed in the 3 L bioreactor for *C. aeruginascens* cultivation (performed with working volume 2 L, ring gasifier, two disc stirrer and three baffle)

Scale‐up criteria	Unit	SmF1	SmF2	SmF3	SmF4
Stirrer speed	[rpm]	800	450	150	0
Aeration rate	[vvm]	0.5	0.5	0.5	0.5
*K*_L_a value	[h^–1^]	114	25	4.5	3.6
Mixing time	[s]	1.4	2.4	6.1	8.9
Tip‐Speed	[m/s]	2.01	1.13	0.38	0
Energy input	[kW/m^3^]	3.44	0.61	0.02	–
Biomass yield	[g/L]	1.04	1.2	1.66	1.3
pH drop ( = start of pigment formation)	[d]	6	6	4	5

**FIGURE 1:**
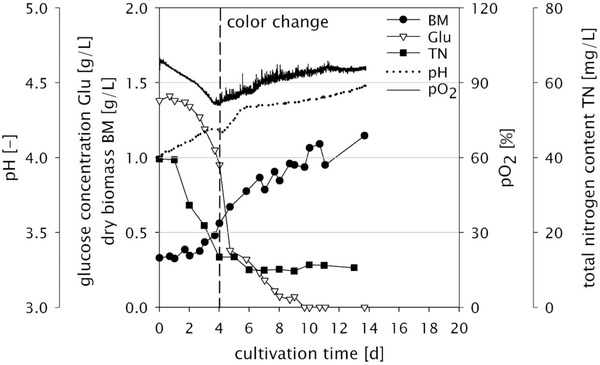
Comparison of the chronological progression of different cultivation parameters and their relationship with regard to the formation of xylindein in a 70 L bioreactor cultivation of *C. aeruginascens* (BM, dry biomass; Glu, glucose concentration; TN, total nitrogen content)

In all experiments, we could exclude oxygen limitation based on the online measurement of the pO_2_ content. An increase of biomass and a decrease of the carbon and nitrogen source over time was detected. The most important parameter to determine optimal fermentation parameters was the production of pigment. Visual observations during cultivations in the bioreactor showed that the onset of pigment production coincides with a lowering of the pH‐value, which starts to increase again after only few hours (Figure S1, see also Figure 1). This temporal pH lowering could function as a primary indicator for successful pigment induction in industrial fermentations. Additionally, this online measurable parameter is independent of visual observations or pigment extraction.

Next to the already mentioned parameters pH and pO_2_, the time course of dry weight (biomass), nitrogen and carbon content were determined. The chronological progression of these parameters is presented in Figure 1 for the largest fermentation (70 L bioreactor), but similar results were obtained in the 3 L screening (compare also with Table 1). As described in our previous work [17], pigment production is triggered by nitrogen limitation. Indeed, in these fermentations the total nitrogen content drops to a constant level at the time of the color change. Since the method detects total nitrogen, even if it is bound in certain molecules, the nitrogen content is not reduced to zero. Therefore, it is assumed that the nitrogen is no longer bioavailable for the organism. At this time between day 4 and 6, the carbon source is still sufficiently available. Of great importance is a suitable uptake of carbon and nitrogen by the fungal biomass and sufficient oxygen supply usually characterized by high *k*_L_a values. An insufficient oxygen supply inhibited the formation of the desired pigment [19]. In the case of *C. aeruginascens*, a high *k*_L_a value is not the crucial parameter for successful cultivation under the given conditions but rather the tip speed as a measure of shear stress, which needs to be moderate to prevent damage to the biomass.

The results showed that at a low stirrer speed (150 rpm, tip speed <0.5 m/s) *C. aeruginascens* had both the fastest pigment formation and the highest biomass concentration. Compared to the three other fermentations with 1.0–1.3 g/L biomass and pigment induction at day 5–6, we achieved the highest biomass concentration of about 1.7 g/L and the earliest start of pigment production at day 4 at 150 rpm (Table 1). Thus, with this screening we could show that too much energy input resulted in reduced biomass yield due to shear stress. This reduction also resulted in a lighter color of the biomass obtained.

Considering the similarity theory, the results of the experiments in the 3 L bioreactor were first transferred to a 7 L bioreactor (working volume 5 L) and then to a 70 L bioreactor (working volume 55 L) (Table 2, Table S1). *K*_L_a values between 4.5 and 7.9 h^–1^ and tip speeds of 0.38 to 0.52 m/s for the different bioreactor volumes were calculated.

**TABLE 2:** Overview of the set scale‐up criteria of the different scales cultivating *C. aeruginascens*

Description	Unit	3 L bioreactor	7 L bioreactor	70 L bioreactor
Working volume	[L]	2	5.5	55
Stirrer speed	[rpm]	150	150	100
Aeration rate	[vvm]	0.5	0.5	0.27
*K*_L_a value	[h^–1^]	4.5	7.9	4.7
Tip‐speed	[m/s]	0.38	0.46	0.52
Energy input	[kW/m^3^]	0.02	0.04	0.02
Biomass yield	[g/L]	1.66	1.44	1.7
pH drop (=start of pigment formation)	[d]	4	4.5	5

As shown in Figure S2, all cultivations show a similar pH‐value curve and biomass concentration. A similar growth rate was also detected for all scales, which was also reflected in the biomass formed per liter of medium used (3 L bioreactor: *μ* = 0.206 d^–1^, Δ*X* = 1.14 g/L; 7 L bioreactor: *μ* = 0.151 d^–1^, Δ*X* = 0.9 g/L; 70 L bioreactor: *μ* = 0.233 d^–1^, Δ*X* = 1.34 g/L). Only minimal delays in pigment formation with increasing working volumes were observed. Therefore, we could show the successfully implemented scale‐up and that the “tip speed,” controlled by the stirrer speed and stirrer geometry, is one of the most important scale‐up criteria in the cultivation of *C. aeruginascens*. Conditions like working volume, aeration rate, and stirrer speed influence the shear stress and have to be optimized to achieve as little damage to the biomass as possible for ideal pigment yield when transferred to industrial scale.

### 3.2 Analysis of xylindein extraction

**FIGURE 2:**
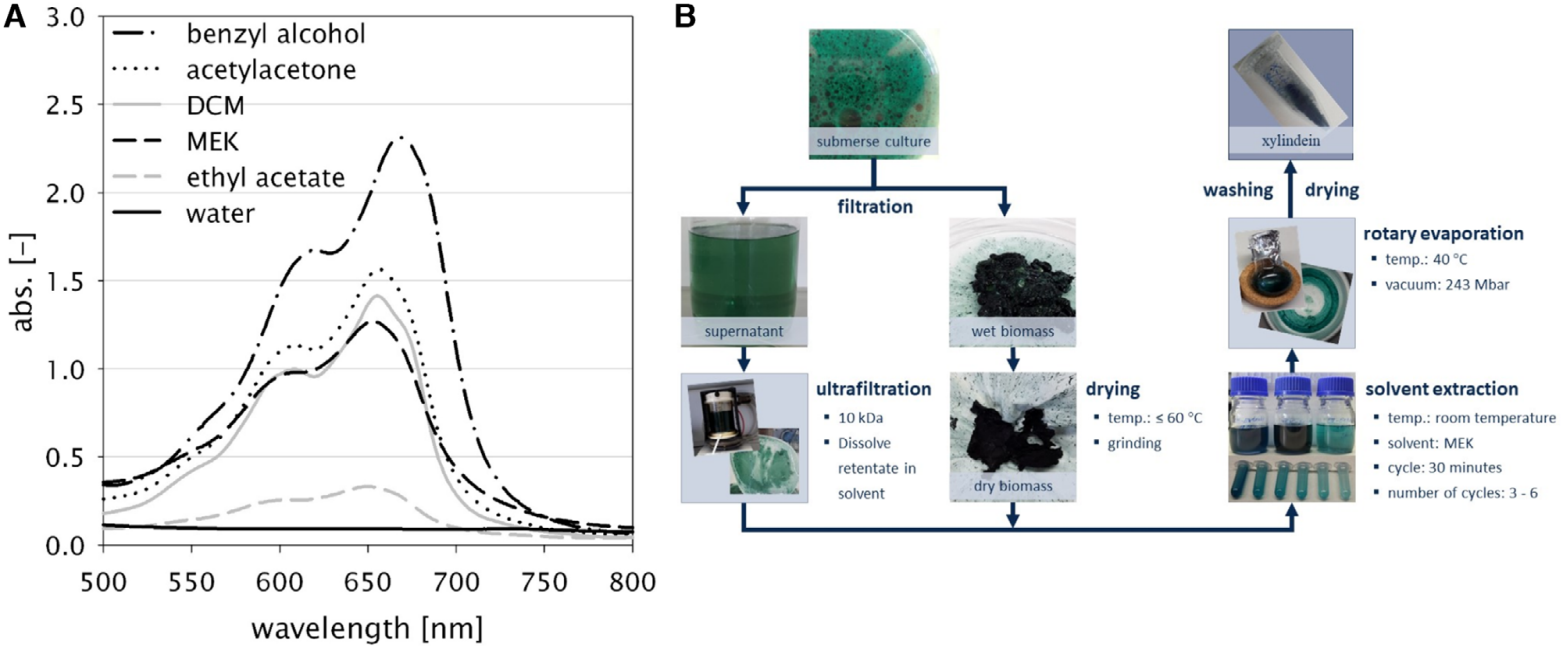
Extraction of xylindein. (A) Absorption spectra of extracts with different solvents and (B) extraction scheme (DCM, dichloromethane, MEK, methyl ethyl ketone)

The pigment xylindein is primarily located in the hyphae and is only released into the environment in small quantities. Cell disruption is therefore of fundamental importance for successful extraction. For this purpose, mechanical treatment, in particular disruption using a high‐pressure homogenizer for fresh biomass and grinding when using dried biomass, has proven to be effective. If fermentation is not directly followed by pigment extraction, dry biomass is easier to handle, for example, for storage and transport. The currently most widely used solvent for xylindein is dichloromethane (DCM) [19, 31] The quantities required for industrial application, however, can only be used under very complex and expensive conditions due to the toxicity of the solvent. This also applies to other solvents suitable for xylindein extraction, for example, chloroform, acetonitrile, or glacial acetic acid [32]. To find an alternative solvent for DCM, an extensive screening was carried out. Solvents of different groups such as hydrocarbons, halogenated hydrocarbons, carboxylic acids, carboxylic acid esters, ketones and diketones, alcohols and alkanols, aromatic organic compounds, carbon‐sulphur compounds, and water were examined. The extracts have a bluish or greenish color depending on the product concentration. Extracts exhibited a characteristic absorption spectrum with two peaks at approx. 610 and 650 nm (Figure 2A), which is identical to values reported in literature [9, 25]. Solvents from the ketone/diketone range, especially acetone, acetylacetone, 2‐butanone (MEK, methyl ethyl ketone), proved to be suitable for extraction. Benzyl alcohol also showed a very good extraction capacity, which was even better than DCM. Due to the later separation of the solvents using rotary evaporation, acetone, ethyl acetate, and MEK, as well as DCM as reference were further investigated. We identified the following parameters as optimal: 30 min extraction time per cycle, a 1:1 ratio of solvent to extraction material, and 3–6 extraction cycles. MEK was found to be the preferred solvent besides DCM. Rotary evaporation produced a dark green powder ready for various applications. For future industrial use, we identified that further processing of the culture supernatant by ultrafiltration (10 kDa or less) allows the recovery of pigment released during cultivation. The complete extraction process is summarized in Figure 2B.

Analysis of the extracts showed that it was free of fat, sugar, and protein. Furthermore, a temperature stability of up to approx. 100°C has been proven. Harrison et al. [23] were even able to report a stability up to 190°C. If the pigment is still bound to the biomass, irreversible damage to the dye (brown coloring) was observed above 60°C. Therefore, if biomass needs to be dried before pigment extraction, it is advised to use low temperature conditions. The extracted pigment (green powder) was stored stably for months, while liquid extract stability depends on the solvent. The DCM extracts were stable for 9 months showing no loss of color, while the MEK extracts increasingly lost intensity, and showed a change in color (blue to pink) after more than 5 months. In general, solvent and product concentration affected color shade and intensity (solvatochromic, hyper‐chromatic, and batho‐chromatic effects).

During the cultivation in the 70 L bioreactor of *C. aeruginascens* in 5% orange juice, approximately 1.3 g/L dry biomass was obtained within 2 weeks. The contained xylindein was extracted by the method developed and described above using MEK (Figure 2B). One gram of dry biomass contained approx. 50 mg xylindein. This in turn corresponds to a product yield of 5%. In comparison, natural dye precursors for indigo production, which can be obtained from dried woad leaves, only resulted in yields of 0.3% [33]. Comparing yields of xylindein, Boonloed et al. [13] achieved 62 mg xylindein from a liquid culture (working volume 250 mL). The biomass concentration was not specified, but the cultivation time was much longer with 10 weeks. Therefore, a productivity of 3.5 mg/L/d of xylindein was achieved. In our study, a productivity of 4.8 mg/L/d was obtained by optimizing the cultivation (medium, parameters) and through the transfer to a much larger scale (working volume 55 L), as well as significant reduction of the cultivation time. The identification of xylindein production resulted in publishing of our patent [34].

### 3.3 Coloration of veneer by fungal surface growth

**FIGURE 3:**
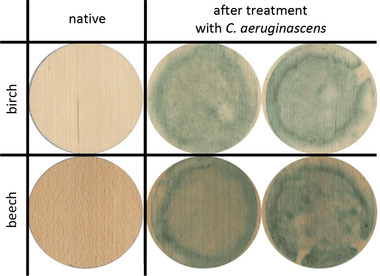
Coloration of veneers with xylindein. Two types of wood have been used for liquid‐solid cultivation of *C. aeruginascens* for a period of 12 weeks

The extracts can be applied to manufactured artwork as done previously by the group around Sara C. Robinson [18]. In our study, we analyzed the coloring of inconspicuous and light types of wood, such as birch or beech, by using of *C. aeruginascens* mycelium cultivation on veneer. We could show that solid wood and veneers were successfully stained by xylindein produced during a combination of solid and liquid cultivation (Figure 3). The most effective staining was achieved by placing wood samples in liquid cultures [35]. Within 12 weeks, surface‐colored solid wood and veneers could thus be produced.

### 3.4 Optimization process for the fermentation of *Laetiporus sulphureus*

**FIGURE 4:**
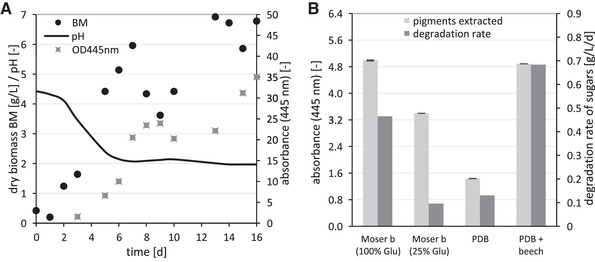
Cultivation of *L. sulphureus* (A) in a bioreactor showing dry biomass, absorption of extract and pH and (B) media optimization in shaking flasks after 14 days of cultivation (BM, dry biomass, Glu, glucose, PDB, potato dextrose broth)

In a screening of 25 different Basidiomycota using submerged cultivation in shaking flasks, we identified *Laetiporus sulphureus* as a potent pigment producer. The pigments have been previously identified as a whole family of different laetiporic acids and have been characterized exhaustively [28]. Compared to other Basidiomycota, biomass growth and pigment production was achieved on a satisfactory level without specific elicitors or tedious media optimization. To optimize pigment generation, we compared the final biomass concentration of *L. sulphureus* cultures from two different media in shaking flasks and analyzed the extracted pigment, which can be identified by the absorbance peak at 445 nm [28]. Compared to standard nutrient liquid (SNL) medium, we achieved a higher pigment to biomass ratio with Moser b medium containing solely glucose as sugar source (data not shown). In both media, most of the pigment is located in the biomass and therefore pigment yield depends on biomass growth. To obtain first insights into the scale up of the cultivation, we utilized Moser b and analyzed biomass growth in a 7 L bioreactor. The bioreactor samples were analyzed for dried biomass and pigment (extract from 1 mL sample), which is shown in Figure 4. After 7 days, a decrease of the determined biomass was observed, which was caused by an inhomogeneous distribution of the biomass in the bioreactor due to floating. At the end of the cultivation, a final probe was taken after harvesting the bioreactor culture. For this purpose, the biomass was mechanically removed from built‐in components and walls and mixed into the liquid phase. Thereby, we achieved a final biomass of 9.54 g/L and an OD_445nm_ = 55.8. Without pH regulation, strong acidification up to pH 2 occurs by the fungus itself, as described previously [29].

Bioeconomy depends on the identification of cost‐effective processes to provide products from sustainable and renewable sources. Compared to complex media like SNL or Moser b, it is therefore advantageous to use cheap and sustainable substrates like agricultural residues or by‐products. We have compared the previously used Moser b and simple media like PDB (potato dextrose broth) with or without additives like ground beech wood. In addition, previous research work ([36], under revision) showed that metabolite pathways may be inhibited by an abundant amount of sugars. Therefore, we tested Moser b with a sugar content of 30 g/L (100%) and 7.5 g/L (25%) glucose and PDB with 10 g/L glucose.

To obtain high nutrient exchange shaking was increased to 170 rpm, which resulted in higher aggregation of the biomass except for cultivations with solid material (Figure S3). The latter induced higher shear forces, resulting in broadly dispersed mycelium and increased nutrient uptake (higher fungal surface). Accordingly, the sugar degradation rate increased with ground beech by 70–80% compared to PDB without beech (Figure 4B), with an almost complete consumption of sugar after 14 days of cultivation. Due to biomass aggregation, the complete culture was homogenized with an ultra‐turrax before sample taking and 2.5 mL sample was extracted with 2.5 mL ethanol (f.c. 50% ethanol). Highest pigment yields were achieved with both Moser b (100% glucose) and PDB with beech. Further investigations, especially regarding optimal growth conditions in bioreactor systems could further improve time‐ and cost‐optimized pigment production. This will focus in particular on the use of sustainable media, known to enhance certain metabolic pathways in Basidiomycota ([36] under revision).

### 3.5 Analysis of laetiporic acids extraction

**FIGURE 5:**
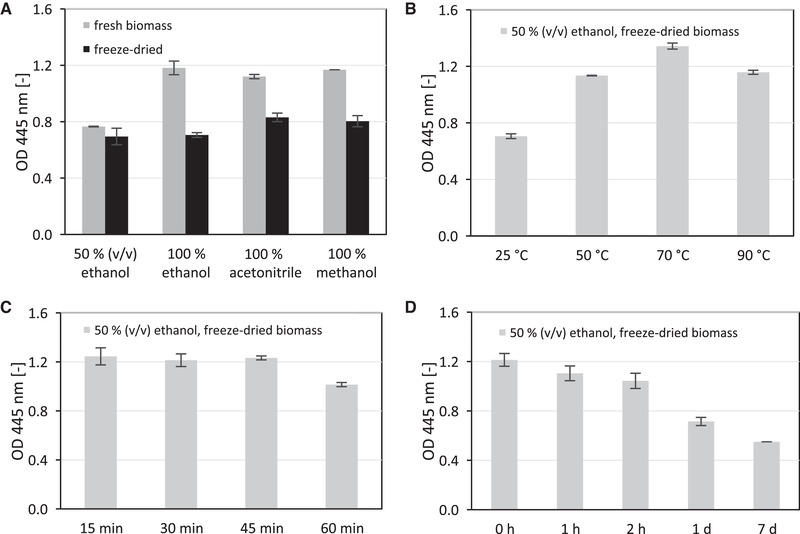
Extraction parameters of laetiporic acids showing the absorption maximum of extracts at OD_445nm_. (A) Extracts with different solvents comparing fresh and freeze‐dried biomass. (B) Comparison of extraction temperature. (C) Comparison of extraction time. (D) Time dependent stability of extract at room temperature

Various solvents were analyzed for their suitability to extract pigment from biomass of *L. sulphureus*. We identified an optimal biomass/solvent ratio of 7.2 g/L freshly harvested biomass in 5 mL solvent (data not shown). This corresponds to a concentration of 0.36 g/L freeze‐dried biomass. Due to problems with dissolving freeze‐dried biomass in the different solutions, we rehydrated the biomass before further treatment. The results for extractions with aqueous ethanol solution (50% (v/v)) and pure ethanol, methanol, and acetonitrile are shown in Figure 5A. Absorption peaked around 445 nm for all solvents (Figure S4A), which is identical to laetiporic acids reported in literature [28]. The extracts contained different laetiporic acid pigments with absorption peaks around 460 nm, as the spectra shows a shoulder at this wavelength (most pronounced with acetonitrile). This was also confirmed by LC‐MS (Figure S4B). Since we achieved very good extraction rates with aqueous ethanol (especially for freeze‐dried samples), extraction temperature and time for this system was further optimized. Further advantages were the reduced solvent consumption and omission of the centrifugation step. We identified an optimal extraction temperature of 70°C (Figure 5B), an optimal extraction time of 15 min (Figure 5C) and determined the extracts’ stability at room temperature to be in the range of a few hours (Figure 5D). The stability increased when aqueous solutions (50% (v/v)) of acetone and acetonitrile were used (Figure S5). For further applications, it is important to determine the color stability at different pH values. According to Stange et al. [19] we performed experiments for the solvents ethanol, acetone, and acetonitrile in buffers at different pH (1:10 ratio). In the acidic and neutral range, no change of absorption was detected. In the alkaline buffer solutions (pH 9–13), an increase in absorption was observed, but this has no effect on the color stability itself (Figure S6). The absorbance peak did not shift, therefore no change in color was detected.

### 3.6 In situ pigment extraction for textile application

**FIGURE 6:**
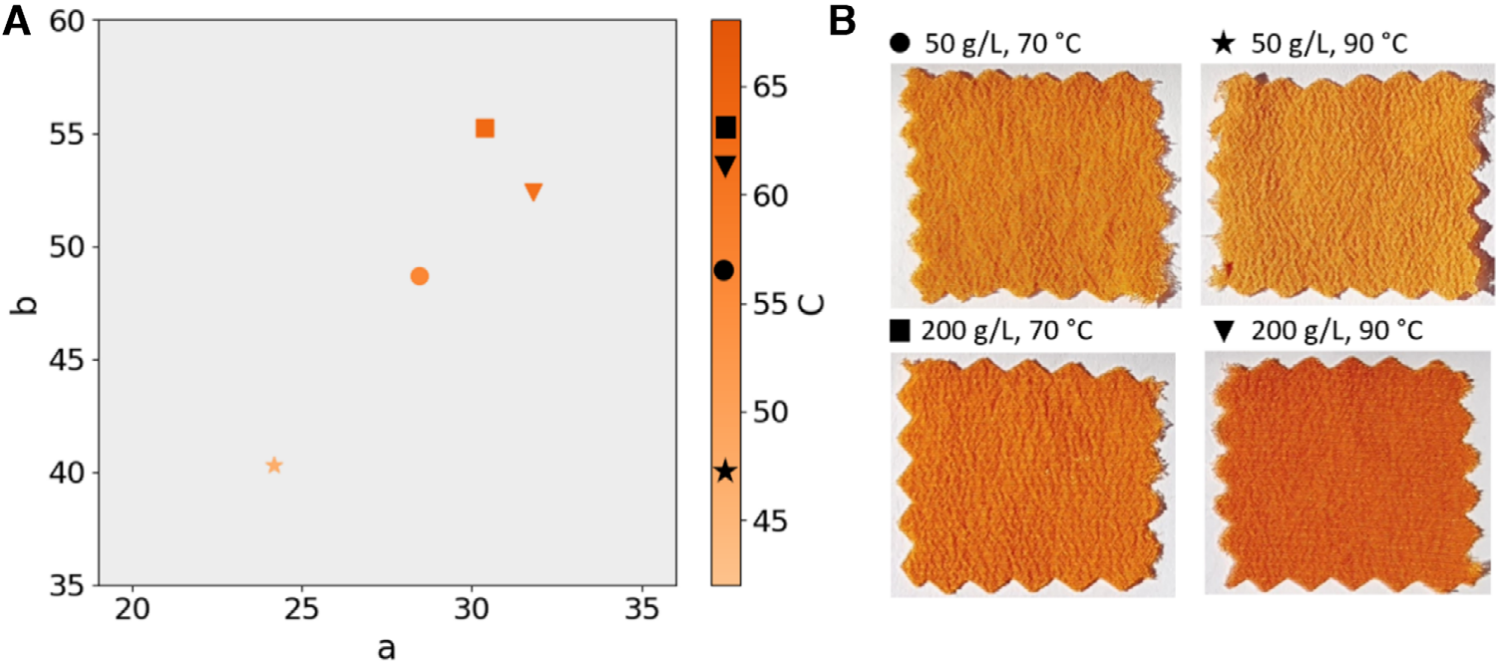
Application of laetiporic acid pigments. (A) CIE lab measurements with the values a, b and C of silk samples dyed with *L. sulphureus* biomass shown in (B). (CIE: International Commission on Illumination; a: color values from green (−) to red (+); b: color values from blue (−) to yellow (+); C: chroma, relative saturation)

Materials like wool and silk can be dyed sustainably using heat to fixate the colorant. Since fruiting bodies of mushrooms have been historically used for dyeing textiles [37], we combined *in situ* extraction with dyeing. In short, freshly harvested biomass was diluted with ethanol (f.c. 50% (v/v)) to achieve biomass concentrations of 50 or 200 g/L. This mixture was immediately used as a dyeing solution during the 60 min long dyeing process. The color intensity increased with increasing biomass (Figure 6A). Increased temperature (90°C instead of 70°C) resulted in an increase of the red value (a) for high biomass concentration. The color of the silk is very brilliant, which is not often seen with natural pigments (Figure 6B).

## 4 CONCLUDING REMARKS

The blue‐green xylindein is a very versatile pigment, which cannot be produced synthetically. So far, it can only be purchased in small quantities through the working group around Sara C. Robinson [26]. Effective biotechnological production is therefore of fundamental importance for industrial use. In our study we were able to successfully scale up our cultivation in the 70 L bioreactor and identify key process parameters, such as the “tip speed” (<0.5 m/s). We were able to reduce the cultivation time significantly to 2 weeks and increase productivity (4.8 mg/L/d). Until now, xylindein has been extracted using DCM. However, DCM can only be used under very complex and expensive conditions due to the toxicity of the solvent. Our extraction optimization has enabled us to identify MEK as a suitable extraction agent for industrial use. Besides the extraction of the pure pigment, the mycological modification of wood by cultivation with *C. aeruginascens* is of great interest, but wood coloring is a long process. Cultivations on solid wood with deeper wood coloring took up to 2.5 years [38]. Additionally, the long‐term cultivations were susceptible to contamination, ineffective coloring, or wood degradation. With our approach using a combination of liquid and solid *C. aeruginascens* cultivation, first results of surface colored wood and veneers were achieved within 3 months.

Next to the blue‐green color of xylindein by *C. aeruginascens*, we investigated the pigment family of laetiporic acids produced by *L. sulphureus*. In our study, upscaling of the pigment production through bioreactor cultivation was achieved. The successful extraction of laetiporic acids with simple and nontoxic solvents and the feasibility to dye silk samples provide a new possibility for natural textile dyeing. In contrast to other fungal pigments mainly produced by mold fungi [2] the toxicity is reduced because *L. sulphureus* is an edible mushroom, known as “chicken of the woods”. Further work will focus on the toxicity of the pigments, as well as evaluation of color fastness, UV resistance, and washing stability. It is also intended to study different materials to extend the application variety.

This study provides more chances for eco‐friendly natural dyes from sustainable resources and offers a solution to counter the hazardous effects of synthetic colorants on the environment and human health.

## CONFLICT OF INTEREST

The authors have declared no conflict of interest.

## FUNDING

This research was funded by the Federal Ministry for Economic Affairs and Energy, grant number KF 2418631 and KF 2049821 and the Federal Ministry of Education and Research, grant number 031B0879.

## DATA AVAILIBILITY STATEMENT

Data available on request from the authors. The data that support the findings of this study are available from the corresponding author upon reasonable request.

## Supporting information



Supplementary information

## References

[1] Murmann, J. H. , Homburg, E. , Comparing evolutionary dynamics across different national settings: the case of the synthetic dye industry, 1857–1914. J. Evol. Econ. 2001, 11, 177–205.

[2] Venil, C. K. , Velmurugan, P. , Dufosse, L. , Devi, P. R. , et al. Fungal pigments: potential coloring compounds for wide ranging applications in textile dyeing. J Fungi 2020, 6, 68. 10.3390/jof6020068 PMC7344934 32443916

[3] Caro, Y. , Venkatachalam, M. , Lebeau, J. , Fouillaud, M. , et al. Pigments and colorants from filamentous fungi. Fungal Metabolites. 2015, 1–70.

[4] Angelini, L. G. , Pistelli, L. , Belloni, P. , Bertoli, A. , et al. *Rubia tinctorum* a source of natural dyes: agronomic evaluation, quantitative analysis of alizarin and industrial assays. Industrial Crops and Products 1997, 6, 303–311.

[5] Yusuf, M. , Shabbir, M. , and Mohammad, F. , Natural colorants: historical, processing and sustainable prospects. Nat Products Bioprospect 2017, 7, 123–145. 10.1007/s13659-017-0119-9 PMC5315675 28093670

[6] Duran, N. , Teixeira, M. F. , De Conti, R. , Esposito, E. , Ecological‐friendly pigments from fungi. Crit. Rev. Food Sci. Nutr. 2002, 42, 53–66. 11837241 10.1080/10408690290825457

[7] Lagashetti, A. C. , Dufosse, L. , Singh, S. K. , Singh, P. N. , Fungal pigments and their prospects in different industries. Microorganisms 2019, 7, 604. 10.3390/microorganisms7120604 PMC6955906 31766735

[8] Seibold, P. S. , Lenz, C. , Gressler, M. , Hoffmeister, D. , The *Laetiporus* polyketide synthase LpaA produces a series of antifungal polyenes. J. Antibiot. 2020, 73, 711–720. 10.1038/s41429-020-00362-6 PMC7473843 32820242

[9] Michaelsen, H. , Unger, A. , Fischer, C.‐H. , Blaugrüne Färbung an Intarsienhölzern des 16. bis 18. Jahrhunderts. Restauro 1992, 98, 17–25.

[10] Blanchette, R. A. , Wilmering, A. M. , Baumeister, M. , The use of green‐stained wood caused by the fungus Chlorociboria in intarsia masterpieces from the 15th century. Holzforschung 1992, 46, 225–232.

[11] Robinson, S. C. , Michaelsen, H. , Robinson, J. C. , Spalted Wood: The History, Science, and Art of A Unique Material. Schiffer Publishing, Limited 2016.

[12] Weber, G. , Chen, H.‐L. , Hinsch, E. , Freitas, S. , et al. Pigments extracted from the wood‐staining fungi *Chlorociboria aeruginosa*, *Scytalidium cuboideum*, and *S. ganodermophthorum* show potential for use as textile dyes. Color. Technol. 2014, 130, 445–452.

[13] Boonloed, A. , Weber, G. L. , Ramzy, K. M. , Dias, V. R. , et al. Centrifugal partition chromatography: A preparative tool for isolation and purification of xylindein from *Chlorociboria aeruginosa* . J. Chromatogr. A 2016, 1478, 19–25. 27919517 10.1016/j.chroma.2016.11.026

[14] Tudor, D. , Margaritescu, S. , Sanchez‐Ramirez, S. , Robinson, S. C. , et al. Morphological and molecular characterization of the two known North American Chlorociboria species and their anamorphs. Fungal biology 2014, 118, 732–742. 25110135 10.1016/j.funbio.2014.05.003

[15] Robinson, S. C. , Tudor, D. , Snider, H. , Cooper, P. A. , Stimulating growth and xylindein production of *Chlorociboria aeruginascens* in agar‐based systems. AMB Express 2012, 2, 15. 22409931 10.1186/2191-0855-2-15 PMC3350399

[16] Van Court, R. C. , Robinson, S. C. , Stimulating production of pigment‐type secondary metabolites from soft rotting wood decay fungi (“Spalting” Fungi), in: Steudler S. , Werner A. , and Cheng J. J. (Eds.), Solid State Fermentation: Research and Industrial Applications, Springer International Publishing, Cham 2019, pp. 109–124. 10.1007/10_2019_93 30891625

[17] Stange, S. , Steudler, S. , Delenk, H. , Werner, A. , et al. Influence of the nutrients on the biomass and pigment production of *Chlorociboria aeruginascens* . J Fungi (Basel) 2019, 5, 40. 10.3390/jof5020040 PMC6617353 31100858

[18] Robinson, S. C. , Hinsch, E. , Weber, G. , Leipus, K. , et al. Wood colorization through pressure treating: the potential of extracted colorants from spalting fungi as a replacement for woodworkers' aniline dyes. Materials 2014, 7, 5427–5437. 28788136 10.3390/ma7085427 PMC5456206

[19] Stange, S. , Steudler, S. , Delenk, H. , Werner, A. , et al. Influence of environmental growth factors on the biomass and pigment production of *Chlorociboria aeruginascens* . J. Fungi 2019, 5, 46. 10.3390/jof5020046 PMC6616924 31181797

[20] Kögl, F. , von Taeuffenbach, G. , Untersuchungen über Pilzfarbstoffe. IV. Über das Xylindein, den Farbstoff des grünfaulen Holzes (I). Justus Liebigs Ann. Chem. 1925, 445, 170–180.

[21] Robinson, S. C. , Gutierrez, S. M. V. , Garcia, R. A. C. , Iroume, N. , et al. Potential for carrying dyes derived from spalting fungi in natural oils. J. Coat. Technol. Res. 2017, 14, 1107–1113.

[22] Robinson, S. C. , Vega Gutierrez , S. M., Garcia, R. A. C. , Iroume, N. , et al. Potential for fungal dyes as colorants in oil and acrylic paints. J. Coat. Technol. Res. 2018, 15, 845–849.

[23] Harrison, R. , Quinn, A. , Weber, G. , Johnson, B. , et al. Fungi‐derived pigments as sustainable organic (opto)electronic materials. Proc. SPIE 2017, 10101, 10101.

[24] Giesbers, G. , Van Schenck, J. , Quinn, A. , Van Court, R. , et al. Xylindein: naturally produced fungal compound for sustainable (opto)electronics. ACS Omega 2019, 4, 13309–13318. 31460459 10.1021/acsomega.9b01490 PMC6704441

[25] Maeda, M. , Yamauchi, T. , Oshima, K. , Shimomura, M. , et al. “Extraction of xylindein from *Chlorociboria aeruginosa* complex and its biological characteristics,” in Technical report of the Technological University of Nagaoka, ed, 2003, pp. 105–111.

[26] www.northernspalting.com. n.d. North. Spalting. URL https://www.northernspalting.com/ (accessed 10.12.20).

[27] Weber, R. W. S. , Mucci, A. , Davoli, P. , Laetiporic acid, a new polyene pigment from the wood‐rotting basidiomycete *Laetiporus sulphureus* (polyporales, fungi). Tetrahedron Lett. 2004, 45, 1075.

[28] Davoli, P. , Mucci, A. , Schenetti, L. , Weber, R. W. , Laetiporic acids, a family of non‐carotenoid polyene pigments from fruit‐bodies and liquid cultures of *Laetiporus sulphureus* (polyporales, fungi). Phytochemistry 2005, 66, 817–23. 15797608 10.1016/j.phytochem.2005.01.023

[29] Hwang, H. S. , Lee, S. H. , Baek, Y. M. , Kim, S. W. , et al. Production of extracellular polysaccharides by submerged mycelial culture of *Laetiporus sulphureus* var. *miniatus* and their insulinotropic properties. Appl. Microbiol. Biotechnol. 2008, 78, 419–429. 18188554 10.1007/s00253-007-1329-6

[30] Miller, G. L. , Use of dinitrosalicylic acid reagent for determination of reducing sugar. Anal. Chem. 1959, 31, 426–428.

[31] Robinson, S. C. , Hinsch, E. , Weber, G. , Freitas, S. , Method of extraction and resolubilisation of pigments from *Chlorociboria aeruginosa* and *Scytalidium cuboideum*, two prolific spalting fungi. Color. Technol. 2014, 130, 221–225.

[32] Regulation (EG) No. 1907/2006 of the European Parliament and of the Council of 18 December 2006 concerning the Registration, Evaluation, Authorisation and Restriction of Chemicals (REACH).

[33] Biertümpfel, A. , Stolte, H. , Wenig, B. , Adam, L. , “Färberpflanzen ‐ Anbau, Farbstoffgewinnung und Färbeeignung. Fachagentur Nachwachsende Rohstoffe e. V.(FNR)”, 2013.

[34] Steudler, S. , Stange, S. , Delenk, H. , Wagenführ, A. , et al. “Verfahren zur biotechnologischen gewinnung des blaugrünen pilzpigments xylindein,” WO2020094552A8, 2020‐07‐02, 2020.

[35] Stange, S. , Steudler, G. F. S. , Delenk, H. , Werner, A. , et al. Optimierung der Pigmentbildung vom holzverfärbenden Pilz Chlorociboria aeruginascens – Teil 1: Biomasse‐ und Pigmentbildung auf Agar und in Flüssigmedien. Holztechnologie 2018, 59, 52–60.

[36] Steinmüller, M. , Zschätzsch, M. , Walther, T. , Werner, A. , Production of laccase by C. unicolor; a critical examination and improvement of established methods including kcat measurements. Eng. Life Sci. 2020. (manuscript in revision).

[37] Tegeler K . Leitfaden zum Färben mit Pilzen. Bayer. Mykolog. Ges., Pegnitz; Auflage: EA 2012 Herg. Von DGfM.

[38] Richter, D. L. , Glaeser, J. A. , Wood decay by *Chlorociboria aeruginascens* (Nyl.) Kanouse (Helotiales, Leotiaceae) and associated basidiomycete fungi. International Biodeterioration & Biodegradation 2015, 105, 239–244.

