# Supplementary material for: Production of natural colorants by liquid fermentation with Chlorociboria aeruginascens and Laetiporus sulphureus and prospective applications
Source: Eng Life Sci. 2021 Jan 26;21(3-4):270–82. doi: 10.1002/elsc.202000079 (PMC7923565; doi:10.1002/elsc.202000079)
Supplement: Supplementary file 1 — Supplementary information [file ELSC-21-270-s001.pdf]

Supporting information (SI)

Production of natural colorants by liquid fermentation with *Chlorociboria aeruginascens* and *Laetiporus sulphureus* and prospective applications

Marlen Zschätzsch[1], Susanne Steudler[2], Olena Reinhardt[1], Pia Bergmann[3], Franziska Ersoy[3], Stephanie Stange[2], André Wagenführ[2], Thomas Walther[1], Ralf Günter Berger[3], Anett Werner[1]

[1]Institute of Natural Materials Technology, Chair of Bioprocess Engineering, Faculty of Mechanical Engineering, Technical University of Dresden, 01069 Dresden, Germany

[2]Institute of Natural Materials Technology, Chair of Wood Technology and Fibre Materials Technology, Technical University of Dresden, 01069 Dresden, Germany

[3]Institute of Food Chemistry, Gottfried Wilhelm Leibniz University Hannover, Callinstraße 5, 30167 Hannover, Germany

Correspondence: Dr. Marlen Zschätzsch, Institute of Natural Materials Technology, Technical University of Dresden, 01069, Dresden, Germany. Email: marlen.zschaetzsch@tu-dresden.de

SI methods

Media composition

<u>SNL</u>

30 g/L glucose monohydrate, 4.5 g/L L-asparagine monohydrate, 3 g/L yeast extract, 1.5 g/L KH_2PO_4; 0.5 g/L $MgSO_4$; 5 µg/L $CuSO_4 \cdot 5\ H_2O$; 80 µg/L $FeCl_3 \cdot 6$ H2O; 30 µg/mL $MnSO_4 \cdot H_2O$; 90 µg/mL $ZnSO_4 \cdot 7\ H_2O$; 400 µg/mL EDTA

<u>Moserb</u>

30 g/L D-glucose· H_2O; 10 g/L malt extract; 2 g/L peptone; 0.15 g/L K_2HPO_4; 0.35 g/L KH_2PO_4; 1 g/L NH_4NO_3; 0.3 g/L $NaNO_3$; 0.5 g/L $MgSO_4 \cdot 7\ H_2O$; 0.1 g/L $CaCl_2$; 0.001 g/L biotin; 0.05 g/L inositol; 0.0018 g/L $ZnSO_4 \cdot 7\ H_2O$; 0.017 g/L $FeCl_3 \cdot 6\ H_2O$; 0.0056 $MnSO_4 \cdot\ H_2O$; 0.05 g/L thiamine.

Purification of culture supernatant

The culture supernatant was filtered using different filter sizes to obtain the xylindein released in the medium. Syringe filter units (0.45 µm) and centrifuge tube filters (10 kDa, 5 kDa) were tested. The tube filters were centrifuged for 10 min at 2600 g. The absorption spectra of the filtrates and starting solutions were recorded.

Determination of extraction cycles

To determine the number of cycles, pretreated fresh biomass was incubated in a ratio of 1:1 with solvent (DCM) for 30 min an overhead shaker, centrifuged (5 min at 2600 g) and the supernatant separated. The pellet was again mixed with 3 mL solvent and incubated for 30 minutes. This cycle was repeated up to 6 times. Afterwards, the absorption spectrum of the supernatants was recorded. For comparison, 10 mg ground dried biomass was mixed with 3 mL each of MEK or DCM and incubated for 30 min in an overhead shaker. The biomass was then centrifuged and separated from the supernatant. For the second extraction cycle, 3 mL of the respective solvent was added to the pellet. This protocol was repeated for up to 7 extraction cycles. Afterwards, the absorption spectra of the supernatants were recorded.

Chromatographic measurement

For LC-MS analysis, approx. 20 mg dried biomass of *L. sulphureus* was extracted with 3·1,5 mL methanol and the extract shaken with 5 mL hexane for 5 min. The methanol phase was dried under nitrogen and the residue taken up using 2 mL acetonitrile. Measurements were performed using a Varian LC-MS/MS system (Pump 212, autosampler 460 and MS 320 with APCI housing; Varian) equipped

with a Nucleodur Pyramid RP column (150/2 mm, 3 µm, Macherey-Nagel, Düren, Germany) at 30 °C. Elution was performed using (A) aqueous 0.1 % formic acid (LC-MS grade, Carl Roth, Karlsruhe, Germany) and (B) acetonitrile with 0.1 % formic acid (LC-MS grade, Carl Roth, Karlsruhe, Germany) with the following gradient: 0 - 5 min 50 % B, 5 – 20 min 50 - 100 % B, 20 - 48 min 100 % B, 48 - 50 min 100 - 50 % B, 50 – 55 min 50 % B at a flow rate of 500 µL /min. Detection occurred using APCI housing 50 °C, drying gas 200 °C, capillary 100 V, collision gas pressure 200 mPa, and detector 1500 V at m/z: SIM: 403, 421, 447, and 473 and scan 300 – 500 in addition to detection at 445 and 460 nm.

SI data

Table S1: Detailed parameters for scale-up from 3 L to 70 L bioreactor.

Description		3 L bioreactor	7 L bioreactor	70 L bioreactor
		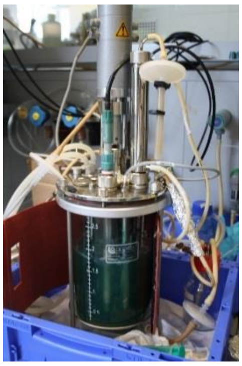	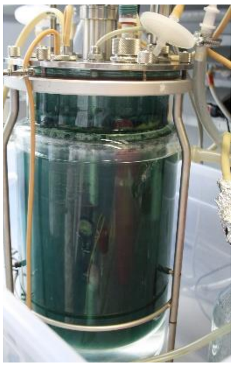	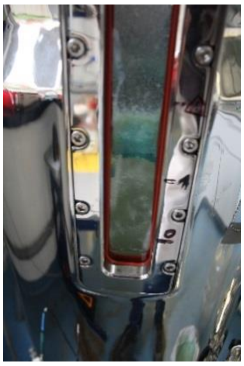
Volume	[L]	3	7	70
Work volume	[L]	2	5.5	55
H/D ratio		1.5	1.8	2.2
Stirrer type		Disc stirrer	Disc stirrer	Disc stirrer
Number of stirrers		2	3	3
Stirrer diameter	[cm]	4.8	4.9	10
Stirrer speed	[rpm]	150	150	100
Installations		3 baffle	3 baffle	4 baffle
Aeration trype		Ring gasifier	Ring gasifier	Ring gasifier
Aeration rate	[vvm]	0.5	0.5	0.27
Temperature control		Heater / heating jacket	Double Jacket	Double Jacket
K_La value	[h^{-1}]	4.5	7.9	4.7
Tip-Speed	[m/s]	0.38	0.46	0.52
Energy input	[kW/m^3]	0.02	0.04	0.02

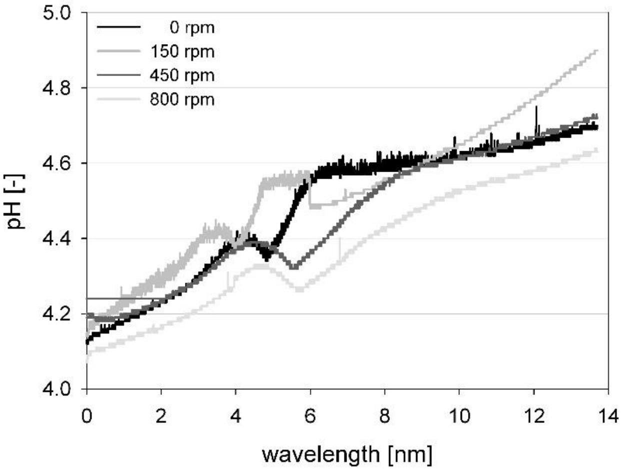

Figure S1: Time course of pH values as a function of the stirrer speed of the C. aeruginascens cultivations in the 3 L bioreactor.

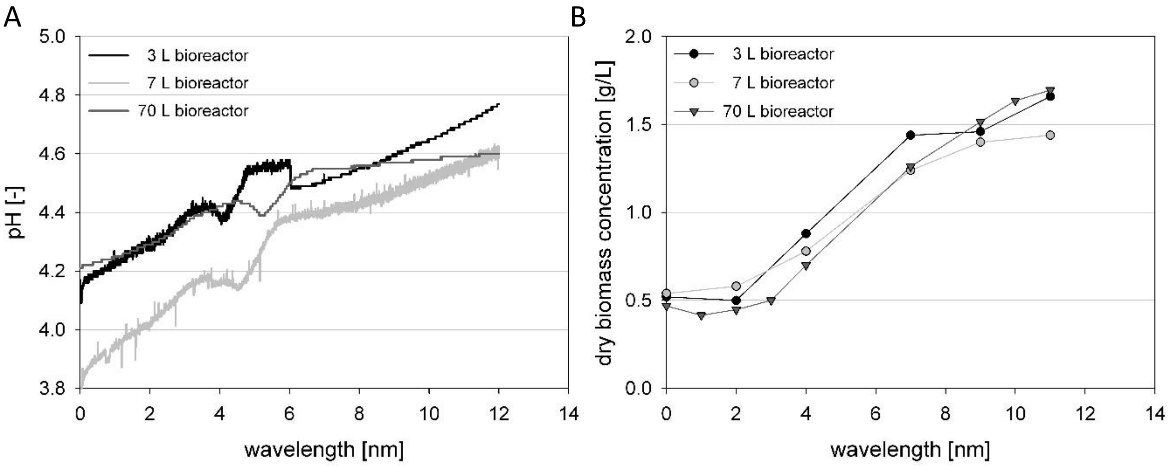

Figure S2: Up scaling bioreactor experiments for C. aeruginascens. A) Time course of pH-values and B) correlating biomass development as a function of cultivation scale.

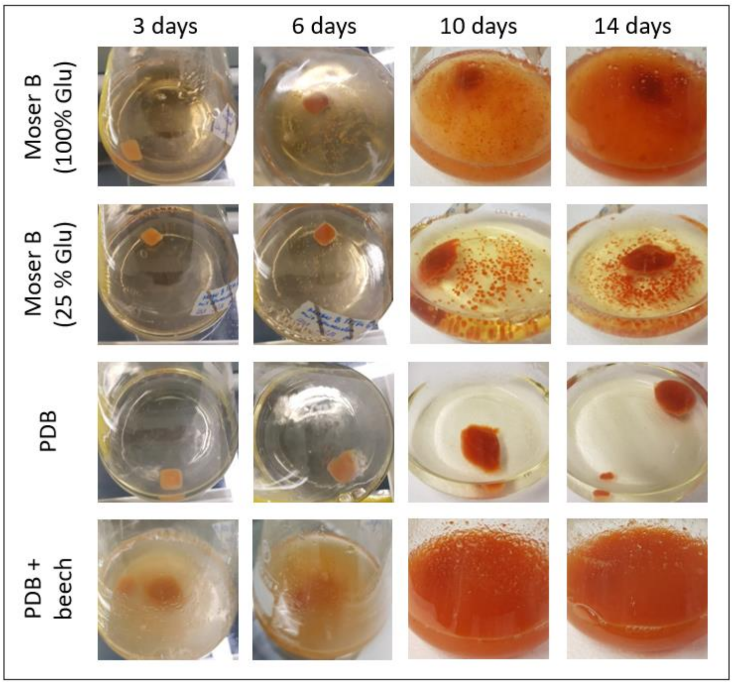

Figure S3: Cultivation of L.sulphureus with different media in shaking flasks. Pictures of the cultures at day 3, 6, 10, and 14.

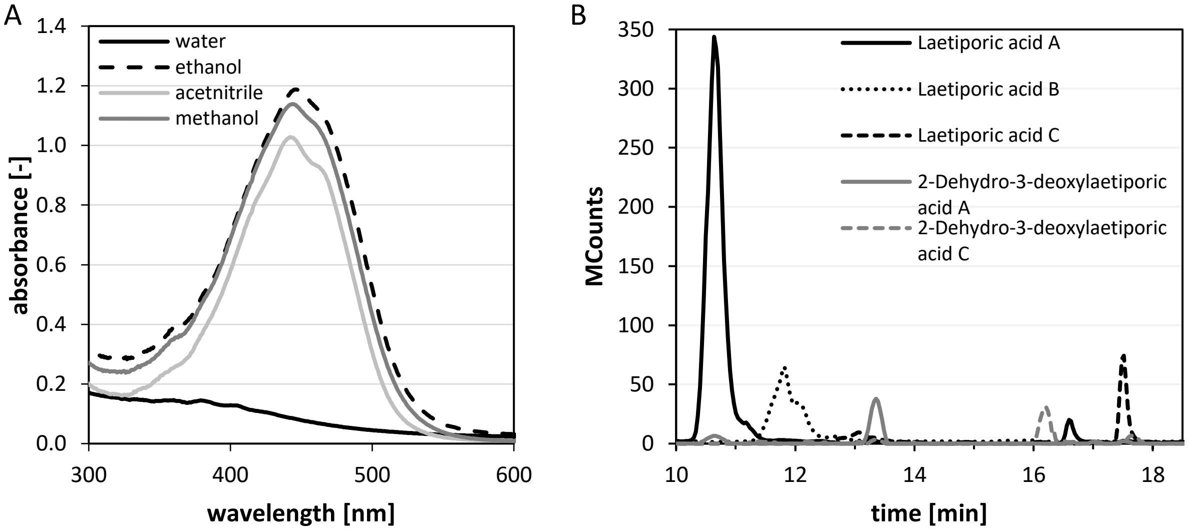

Figure S4: Spectrophotometric and chromatographic analysis of pigment extracts. A) Absorption spectra of extracts with various solvents from freeze-dried LSU biomass. B) LC-MS analysis of the methanol extract of the dried biomass. Shown are the selected ion masses of the different laetiporic acids as described by Davoli et al.*[20].*

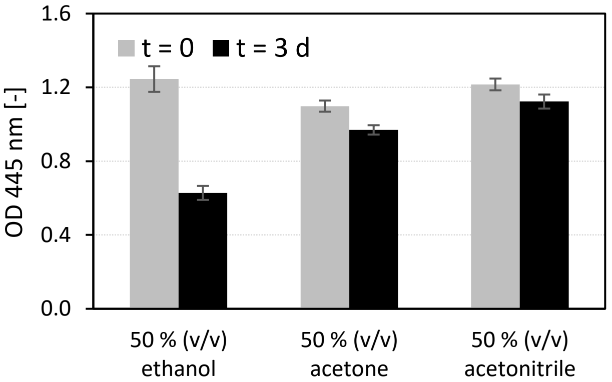

Figure S5: Time dependent stability of extracts using various aqueous solvent solutions. Extract remained for three days at room temperature.

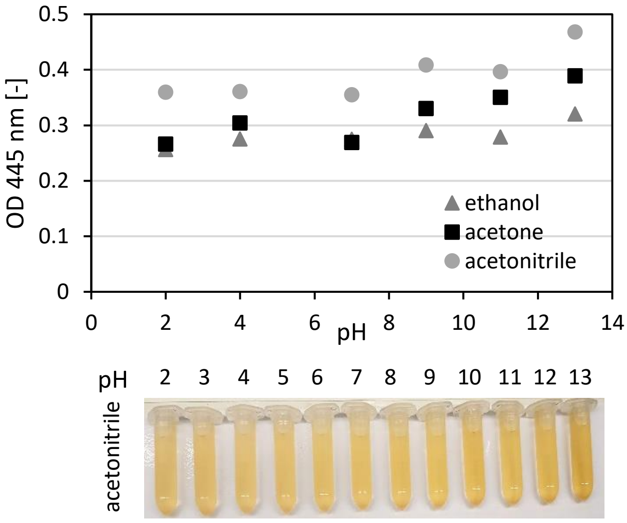

Figure S6: Color stability of laetiporic acid at different pH values (1:10 dilution of extract in buffer with certain pH).
